# Estimation of 1-Year Changes in Medicaid Expenditures Associated With Reducing Cigarette Smoking Prevalence by 1%

**DOI:** 10.1001/jamanetworkopen.2019.2307

**Published:** 2019-04-12

**Authors:** Stanton A. Glantz

**Affiliations:** 1 Center for Tobacco Control Research and Education, Philip R. Lee Institute for Health Policy Studies, Department of Medicine, University of California, San Francisco

## Abstract

**Importance:**

Reducing smoking is associated with a reduction in health care costs, including in the short run. Medicaid recipients smoke at higher rates than the general population, which suggests that investments to reduce smoking in this population would reduce short-run Medicaid costs.

**Objective:**

To estimate the short-run (1-year) change in health care expenditure associated with a 1% decrease in absolute smoking prevalence in all US states.

**Design, Setting, and Participants:**

Economic evaluation based on state Medicaid expenditures and the elasticity between changes in smoking prevalence and health care costs. Data sources were the 2017 Behavioral Risk Factors Surveillance System, 2017 National Health Interview Survey, and Kaiser Family Foundation Total Medicaid Spending for fiscal year 2017. Analysis was conducted in 2018. Participants were all people receiving Medicaid in all US states and the District of Columbia.

**Exposures:**

Cigarette smoking.

**Main Outcomes and Measures:**

Short-run (1-year) change in health care costs.

**Results:**

Reducing absolute smoking prevalence by 1% in each state was associated with substantial Medicaid savings the following year, totaling $2.6 billion (in 2017 dollars). Each state saved a median (interquartile range) of $25 million ($8 million to $35 million).

**Conclusions and Relevance:**

Effective efforts to reducing smoking could be a cost-effective way to reduce Medicaid costs in the short run.

## Key Points

**Question:**

What are the expected changes to Medicaid costs the following year associated with reducing absolute cigarette smoking prevalence in each state by 1%?

**Findings:**

This economic evaluation found that, based on observed short-run elasticity between changes in smoking and health care expenditures, estimated Medicaid savings in the year following a 1% reduction of smoking prevalence would total $2.6 billion, with median state savings of $25 million.

**Meaning:**

Reducing cigarette smoking could contribute to lowering Medicaid costs in the short run.

## Teaser

This economic evaluation estimates the 1-year changes in US Medicaid expenditures associated with a 1% reduction in cigarette smoking prevalence.

## Introduction

In fiscal year 2017, total Medicaid costs were $577 billion.^1^ It is widely accepted that reducing smoking is associated with a reduction in health care costs, but the implicit assumption has been that it takes years to see these savings. While this may be true for some diseases, notably cancer, other risks change quickly in response to changes in smoking behavior, including myocardial infarction, lung disease, and complications of pregnancy. Medicaid recipients smoke at higher rates than the general population; in 2017, 24.5% of adult Medicaid recipients (aged ≥18 years) smoked cigarettes compared with 14.0% of all adults,^2^ suggesting that investments to reduce smoking in this population could be associated with a reduction in Medicaid costs in the short run.^3^

## Methods

This article reports the results of a secondary data analysis using large deidentified data sets, which is not considered human subjects research and is therefore exempt from review according to the University of California, San Francisco, institutional review board. Reporting of the study followed the Consolidated Health Economic Evaluation Reporting Standards (CHEERS) reporting guideline. The analysis was conducted in 2018.

### Study Population and Data Sources

Participants of the study were people receiving Medicaid in all US states and the District of Columbia. Data sources were the 2017 Behavioral Risk Factors Surveillance System, 2017 National Health Interview Survey (NHIS), and Kaiser Family Foundation Total Medicaid Spending for fiscal year 2017. The 2017 Behavioral Risk Factors Surveillance System provides smoking prevalence for each state^4^ but does not provide data on smoking prevalence for people receiving Medicaid. In contrast, the 2017 NHIS does identify Medicaid recipients,^5^ defined as persons who do not have private coverage, but who have Medicaid or other state-sponsored health plans including the Children’s Health Insurance Program. The NHIS does not provide state-level data; it aggregates the states into 4 major census regions (Northeast, Midwest, South, and West) (Table 1).

**Table 1.** Cigarette Smoking Prevalence Among All Adults and Medicaid Recipients^a^

Region^b^	All Adults, %	Medicaid Recipients, %	Ratio
Northeast	11.2	23.8	2.12
Midwest	16.9	36.1	2.14
South	15.5	27.6	1.78
West	11.0	15.1	1.38
All	14.0	24.5	1.76

^a^ The sample size was 26 630 less 90 missing people for the Medicaid estimates because their insurance was not reported.

^b^ The Northeast region includes Connecticut, Maine, Massachusetts, New Hampshire, New Jersey, New York, Pennsylvania, Rhode Island, and Vermont; the Midwest includes Illinois, Indiana, Iowa, Kansas, Michigan, Minnesota, Missouri, Nebraska, North Dakota, Ohio, South Dakota, and Wisconsin; the South includes Alabama, Arkansas, Delaware, District of Columbia, Florida, Georgia, Kentucky, Louisiana, Maryland, Mississippi, North Carolina, Oklahoma, South Carolina, Tennessee, Texas, Virginia, and West Virginia; and the West includes Alaska, Arizona, California, Colorado, Hawaii, Idaho, Montana, Nevada, New Mexico, Oregon, Utah, Washington, and Wyoming.

### Statistical Analysis

Adult Medicaid recipients smoke (defined as persons who had smoked ≥100 cigarettes during their lifetime and now smoked cigarettes either every day or some days) at higher rates than the general population. In addition, there are differences in cigarette smoking by Medicaid recipients across states as well as differences in Medicaid eligibility, benefits, and medical costs.

We used the NHIS to estimate the cigarette smoking prevalence among the general population and Medicaid recipients in each census region accounting for person-level weights (Table 1), then computed the ratio of these 2 prevalences (Table 1). We then multiplied the Behavioral Risk Factors Surveillance System prevalence estimates for each state (Table 2, third column) in each of the 4 census regions (Table 2, second column) by the corresponding ratio (Table 2, fifth column) for states in that census region to estimate cigarette smoking prevalence among Medicaid recipients in each state (Table 2, fourth column).

**Table 2.** Predicted Reductions in State Medicaid Costs the Year Following a 1% Decrease in Absolute Cigarette Smoking Prevalence (2017)

State	Region	General Population Cigarette Smoking Prevalence, %	Medicaid Cigarette Smoking Prevalence, %	Ratio of Smoking Prevalence Between Medicaid and General Population	Relative Decrease in Prevalence Corresponding to 1% Decrease in Absolute Prevalence, %	Medicaid Cost, $, Million	Estimated Reduction in Medicaid Costs the Following Year, $, Million
Alaska	W	21.0	28.9	1.38	3.5	5593	22.8
Alabama	S	20.9	37.2	1.78	2.7	1979	6.3
Arkansas	S	22.3	39.7	1.78	2.5	11 826	35.2
Arizona	W	15.6	21.5	1.38	4.7	6423	35.3
California	W	11.3	15.5	1.38	6.4	83 033	630.2
Colorado	W	14.6	20.1	1.38	5.0	7876	46.3
Connecticut	NE	12.7	26.9	2.12	3.7	7966	34.9
District of Columbia	S	14.4	25.6	1.78	3.9	2142	9.9
Delaware	S	17.0	30.2	1.78	3.3	2806	10.9
Florida	S	16.1	28.6	1.78	3.5	23 281	95.9
Georgia	S	17.5	31.1	1.78	3.2	10 204	38.7
Hawaii	W	12.8	17.6	1.38	5.7	2387	16.0
Iowa	M	17.1	36.6	2.14	2.7	1842	5.9
Idaho	W	14.4	19.8	1.38	5.0	15 151	90.2
Illinois	M	15.5	33.1	2.14	3.0	11 167	39.8
Indiana	M	21.8	46.6	2.14	2.1	4157	10.5
Kansas	M	17.4	37.2	2.14	2.7	3234	10.3
Kentucky	S	24.6	43.8	1.78	2.3	9586	25.9
Louisiana	S	23.1	41.1	1.78	2.4	11 038	31.7
Massachusetts	NE	13.7	29.0	2.12	3.4	2683	10.9
Maryland	S	13.9	24.7	1.78	4.0	11 231	53.6
Maine	NE	17.3	36.7	2.12	2.7	17 279	55.6
Michigan	M	19.3	41.3	2.14	2.4	16 828	48.1
Minnesota	M	14.5	31.0	2.14	3.2	11 483	43.7
Missouri	M	20.8	44.5	2.14	2.2	5479	14.5
Mississippi	S	22.2	39.5	1.78	2.5	10 151	30.3
Montana	W	17.2	23.7	1.38	4.2	1792	8.9
North Carolina	S	17.2	30.6	1.78	3.3	2082	8.0
North Dakota	M	18.3	39.1	2.14	2.6	3557	10.7
Nebraska	M	15.4	32.9	2.14	3.0	2072	7.4
New Hampshire	NE	15.7	33.3	2.12	3.0	14 956	53.0
New Jersey	NE	13.7	29.0	2.12	3.4	4828	19.6
New Mexico	W	17.5	24.1	1.38	4.2	77 822	381.4
Nevada	W	17.6	24.2	1.38	4.1	13 532	65.9
New York	NE	14.1	29.9	2.12	3.3	1222	4.8
Ohio	M	21.1	45.1	2.14	2.2	23 162	60.6
Oklahoma	S	20.2	35.9	1.78	2.8	4966	16.3
Oregon	W	16.1	22.2	1.38	4.5	8387	44.7
Pennsylvania	NE	18.8	39.9	2.12	2.5	28 279	83.7
Rhode Island	NE	15.0	31.8	2.12	3.1	2637	9.8
South Carolina	S	18.8	33.4	1.78	3.0	6204	21.9
South Dakota	M	19.3	41.3	2.14	2.4	860	2.5
Tennessee	S	22.6	40.2	1.78	2.5	9138	26.8
Texas	S	15.7	27.9	1.78	3.6	36 344	153.6
Utah	W	8.9	12.2	1.38	8.2	2509	24.2
Virginia	S	16.4	29.2	1.78	3.4	1601	6.5
Vermont	NE	15.8	33.5	2.12	3.0	9044	31.9
Washington	W	13.5	18.6	1.38	5.4	12 006	76.3
Wisconsin	M	16.0	34.2	2.14	2.9	4037	13.9
West Virginia	S	26.0	46.2	1.78	2.2	8174	20.9
Wyoming	W	18.7	25.7	1.38	3.9	599	2.7
All states	NA	14.0	24.5	NA	NA	576 638	2609.5

Abbreviations: M, Midwest; NA, not applicable; NE, Northeast; S, South; W, West.

Lightwood and Glantz^6^ quantified short-run changes in health care costs the year after changes in smoking behavior and found that 1% relative reductions in current smoking prevalence and mean packs smoked per current smoker are associated with mean (SE) reductions of 0.118% (0.026%) and 0.108% (0.025%) in per capita health care expenditure (elasticities). For this analysis, we concentrate on changes in prevalence. (Given the similar elasticity for changes in consumption per smoker, the results would be similar for changes in consumption per smoker. One could also estimate the effects of simultaneous changes in both variables by adding the effects of the 2 changes.)

To apply the 0.118 elasticity between changes in cigarette smoking prevalence and changes in health care expenditures the following year, we need to compute the relative decrease in prevalence that corresponds to a 1% decrease in absolute prevalence in each state. Dividing the 1% absolute prevalence decrease by the estimated Medicaid prevalence (Table 2, fourth column) yields the corresponding relative change in prevalence (Table 2, sixth column).

Finally, we multiplied the state Medicaid cost for 2017 reported by the Kaiser Family Foundation^1^ (Table 2, seventh column) by the 0.118 elasticity and the relative change in prevalence (Table 2, sixth column) to obtain the estimated Medicaid savings in each state (Table 2, eighth column) if they lowered absolute cigarette smoking prevalence by 1%.

## Results

Table 2 shows that reducing absolute smoking prevalence by 1% in each state was associated with substantial Medicaid savings the following year, totaling $2.6 billion (in 2017 dollars). The median (interquartile range) state savings was $25 million ($8 million to $35 million).

## Discussion

The results of this study indicate that investments in policies to motivate and assist Medicaid recipients to stop smoking may yield substantial savings in short-term medical costs.

These estimates are based on cigarette smoking only,^6^ and the use of noncigarette tobacco products is increasing. While 14.0% of adults were current cigarette smokers in 2017, 19.3% used some tobacco product.^2^ To the extent that tobacco control programs are associated with reduced use of these other tobacco products, there could be additional savings. This analysis is focused on reductions in prevalence; reductions in consumption by continuing smokers may also be followed by reductions in health care costs.^6^ Because some of the risks of smoking, such as cancer, emerge more slowly over time, these medical cost savings would likely grow with time.

### Limitations

The fact that the NHIS data are only available by region but are applied to individual states introduces uncertainty, as different states have different age, racial, and sex distributions. Unfortunately, more granular data are not available. Many Medicaid recipients are children (and so their health costs are included in Table 2, seventh column), but the estimates of Medicaid savings are based only on adult smoking. Because the elasticity estimates are based on aggregate measures of population characteristics, these estimates reflect all the health care expenditures associated with smoking that arise in a population, which include at least some of the indirect health effects on smokers and of secondhand and thirdhand smoking exposure to nonsmokers, including children.^6^

## Conclusions

In addition to the benefits in terms of improved health, this study suggests that reducing smoking among Medicaid recipients may result in substantial savings to the Medicaid program, which would release these funds for other state and federal priorities.
