# Error in Key Points, Abstract, Results Section, and Table

**DOI:** 10.1001/jamanetworkopen.2019.8599

**Published:** 2019-07-31

In the Original Investigation titled “Estimation of 1-Year Changes in Medicaid Expenditures Associated With Reducing Cigarette Smoking Prevalence by 1%,”^1^ published April 12, 2019, there were errors in the estimated change in expenditures overall and by state in the Key Points, Abstract, Results section, and Table 2. The overall savings should have been $2.5 billion and the median (range) state savings, $26 million ($10 million to $52 million). This article has been corrected.^1^

## References

[1] Glantz SA Estimation of 1-year changes in Medicaid expenditures associated with reducing cigarette smoking prevalence by 1%. JAMA Netw Open. 2019;2(4):. doi:10.1001/jamanetworkopen.2019.2307 30977860 PMC6481435

